# Microaerobic enrichment of benzene-degrading bacteria and description of *Ideonella benzenivorans* sp. nov., capable of degrading benzene, toluene and ethylbenzene under microaerobic conditions

**DOI:** 10.1007/s10482-022-01759-z

**Published:** 2022-07-16

**Authors:** Anna Bedics, András Táncsics, Erika Tóth, Sinchan Banerjee, Péter Harkai, Balázs Kovács, Károly Bóka, Balázs Kriszt

**Affiliations:** 1 Department of Molecular Ecology, Institute of Aquaculture and Environmental Safety, Hungarian University of Agriculture and Life Sciences, Páter K. u. 1, Gödöllő, H-2100 Hungary; 2 Department of Microbiology, Eötvös Loránd University, Budapest, Hungary; 3 Department of Environmental Safety, Institute of Aquaculture and Environmental Safety, Hungarian University of Agriculture and Life Sciences, Gödöllő, Hungary; 4 Department of Plant Anatomy, Eötvös Loránd University, Budapest, Hungary

**Keywords:** Benzene, Biodegradation, Comamonadaceae, Ideonella

## Abstract

In the present study, the bacterial community structure of enrichment cultures degrading benzene under microaerobic conditions was investigated through culturing and 16S rRNA gene Illumina amplicon sequencing. Enrichments were dominated by members of the genus *Rhodoferax* followed by *Pseudomonas* and *Acidovorax*. Additionally, a pale amber-coloured, motile, Gram-stain-negative bacterium, designated B7^T^ was isolated from the microaerobic benzene-degrading enrichment cultures and characterized using a polyphasic approach to determine its taxonomic position. The 16S rRNA gene and whole genome-based phylogenetic analyses revealed that strain B7^T^ formed a lineage within the family Comamonadaceae, clustered as a member of the genus *Ideonella* and most closely related to *Ideonella dechloratans* CCUG 30977^T^. The sole respiratory quinone is ubiquinone-8. The major fatty acids are C_16:0_ and summed feature 3 (C_16:1_ *ω*7c/iso-C_15:0_ 2-OH). The DNA G + C content of the type strain is 68.8 mol%. The orthologous average nucleotide identity (OrthoANI) and *in silico* DNA–DNA hybridization (dDDH) relatedness values between strain B7^T^ and closest relatives were below the threshold values for species demarcation. The genome of strain B7^T^, which is approximately 4.5 Mb, contains a phenol degradation gene cluster, encoding a multicomponent phenol hydroxylase (mPH) together with a complete *meta*-cleavage pathway including a I.2.C-type catechol 2,3-dioxygenase (*C23O*) gene. As predicted by the genome, the type strain is involved in aromatic hydrocarbon-degradation: benzene, toluene and ethylbenzene are degraded aerobically and also microaerobically as sole source of carbon and energy. Based on phenotypic characteristics and phylogenetic analysis, strain B7^T^ is a member of the genus *Ideonella* and represents a novel species for which the name *Ideonella benzenivorans* sp. nov. is proposed. The type strain of the species is strain B7^T^ (= LMG 32,345^T^ = NCAIM B.02664^T^).

**Supplementary Information:**

The online version contains supplementary material available at 10.1007/s10482-022-01759-z.

## Introduction

The increasing level of petroleum hydrocarbon pollution significantly damages the ecosystem or even the human health. Simple aromatic hydrocarbons such as benzene, toluene, ethylbenzene, and xylene (BTEX) are the most common contaminants of the groundwater and can therefore easily contaminate drinking water due to their relatively high water solubility. Benzene is an important industrial chemical (Conte et al. 2021) and due to the discharge or disposal of product, benzene can pass into air, water, and soil as well. Accidental leakage of the underground storage tanks release benzene into water and soil where it breaks down more slowly than in air. Exposure to benzene can occur through the lungs, gastrointestinal tract, and across the skin (Abdel-Rahman et al. 1988; da Poça et al. 2021). Benzene can damage the blood and the immune system and can cause cancer such as acute myeloid leukemia and its genotoxic properties are also known (Whysner 2000; Natelson 2007; Guo et al. 2021; da Poça et al. 2021; Wang et al. 2021). Aromatic hydrocarbons decompose most rapidly and completely under aerobic conditions and the bioremediation has proven to be the most successful in solving problems of a widespread contamination. In bioremediation procedures, bacteria are used to eliminate petroleum hydrocarbons as a source of carbon and energy for their metabolic processes. Due to the presence of aerobic microorganisms the concentration of dissolved oxygen in the contaminated soil decreases rapidly partly because the BTEX degraders use mono- and dioxygenases for the hydroxylation and the cleavage of the aromatic ring and these enzymes require molecular oxygen as a co-substrate (Peréz-Pantoja et al. 2019). The availability of dissolved oxygen has a key role in the biodegradation because benzene, *para*- and *ortho*-xylene can be persistent contaminants under anaerobic conditions. Several research suggest that microbial communities in oxygen-limited, aromatic hydrocarbon-contaminated subsurface are generally dominated by members of the Betaproteobacteriales, including genera of the Comamonadaceae family (Fahy et al. 2006; Nestler et al. 2007; Martínez-Lavanchy et al. 2015). Enzymes belonging to the subfamily I.2.C of extradiol dioxygenases are adapted to low oxygen concentrations resulting a large diversity of catechol 2,3-dioxygenase (C23O) genes in these environments. These *C23O* genes are mostly associated with Betaproteobacteriales bacteria (Táncsics et al. 2010, 2012, 2013), and some of them are evidently involved in the microaerobic degradation of BTEXs, e.g. in case of the toluene-degrader *Zoogloea oleivorans* (Táncsics et al. 2020) or *Variovorax paradoxus*, which was capable of degrading all the six BTEX-compounds under both aerobic and microaerobic conditions (Benedek et al. 2021). However, still little is known about the role of microorganisms and I.2.C-type *C23O* genes in microaerobic benzene degradation, since only handful of known cultured bacteria are available yet for the purpose. Consequently, exploration of the bacterial communities of aromatic hydrocarbon contaminated, hypoxic environments, and investigation of bacteria and their functional genes that can participate in biodegradation of benzene and other BTEX components under microaerobic/hypoxic conditions have a current importance.

The genus *Ideonella* was first proposed by Malmqvist et al. (1994) with the description of *Ideonella dechloratans*, which was isolated from activated sludge from a municipal wastewater treatment plant. A significant emendation of the genus was carried out by Noar and Buckley (2009) with the description of *Ideonella azotifigens* isolated from grass rhizosphere soil. To date, five species of the genus *Ideonella* with validly published names have been reported (https://lpsn.dsmz.de/genus/ideonella). The genus *Ideonella* is a member of the family Comamonadaceae belonging to the *Rubrivivax*–*Roseateles*–*Leptothrix*–*Azohydromonas*–*Aquincola*–*Ideonella* branch of the order Burkholderiales (Garrity et al. 2005). The family Comamonadaceae includes several genera involved in the biodegradation of petroleum hydrocarbons as dominant community members in contaminated subsurface environments (Benedek et al. 2018; Révész et al. 2020a,b; Banerjee et al. 2021). Furthermore, they showed a possession of diverse *C230* genes in such environments (Táncsics et al. 2010, 2012, 2013). Members of the genus *Ideonella* are characterized as Gram-stain-negative, rod-shaped bacteria, which contain ubiquinone-8 (Q-8) as predominant isoprenoid quinone; C_16:0_ and summed feature 3 (comprising C_16 : 1_ ω7*c* and/or C_16 : 1_ ω6*c*) as major fatty acids and the G + C content of the genomic DNA is between 67.4 and 70.4 mol% (Malmqvist et al. 1994; Noar and Buckley 2009; Sheu et al. 2016; Tanasupawat et al. 2016; Chen et al. 2020). In this study, a novel species of the genus *Ideonella* isolated from a microaerobic benzene-degrading enrichment culture having BTEX-degrading capability has been described with polyphasic characterization. In addition, whole-genome analysis of strain *Ideonella* designated B7^T^ has been explored providing deeper insights into the genetic background of its BTEX-degrading capability.

## Materials and methods

### Experimental design of enrichment culturing

To reach the goals of the present study, microaerobic benzene-degrading enrichment cultures were set up. All three replicates of the enrichment cultures were inoculated with petroleum hydrocarbon contaminated groundwater of the “Siklos” site of Hungary, (45° 51 × 25.8˝ N 18° 17 × 32.3˝ E) (Táncsics et al. 2012). This area is one of the best characterized BTEX (benzene, toluene, ethylbenzene, and xylenes) contaminated environments of the country, located in Southwest-Hungary. The site has been thoroughly studied and described by Táncsics et al. earlier (2012, 2013). In the course of experiment 100-mL serum bottles were used, sealed with butyl-rubber septa and aluminium crimp seals. To assemble the enrichment cultures, 45 mL of a mineral salt medium (MS) (Fahy et al. 2006) supplemented with vitamins (1 mg thiamine l^− 1^, 15 µg biotin l^− 1^ and 20 µg vitamin B12 l^− 1^) was used for each bottle. The enrichment medium was sparged with N_2_/CO_2_ (80:20, v/v) for 10 min and then sterile air (0.2 μm pore size filtered) was injected through the septa to set the 0.5 mg L^− 1^ concentration of dissolved oxygen. Microaerobic condition was monitored non-invasively by Fibox 3 trace v3 fibre optic oxygen meter with PSt3 sensor spots (PreSens). In addition, each enrichment culture contained 5 mL groundwater sample and benzene as sole carbon and energy source at a concentration of 1 mM. To maintain the microaerobic conditions the oxygen was continuously replenished in the enrichments. After 1 week incubation in shaking thermostat (28 °C, 150 rpm) 5 mL of each enrichment was inoculated into fresh MS medium. The transfer was performed for five consecutive weeks. During the 5th week of the enrichment process, the concentration of benzene was detected for every 24 h by headspace analysis on an ISQ Single Quadrupole gas chromatography-mass spectrometer (GC–MS) (Thermo Fischer Scientific) via an SLB-5 ms fused silica capillary column (Supelco Analytical). The oven temperature was set to 40 °C for 3 min, then ramped at a rate of 20 ºC min ^− 1^ to 190 °C, and held for 1 min. The mass spectrometer was operated at 250 °C in full scan mode.

### 16S rRNA gene amplicon sequencing and data handling

After the 5th transfer, 45 mL of the enrichments was centrifuged at 2360 *g* at 4 °C for 10 min using a Rotanta 460 R centrifuge (Hettich) to harvest the bacterial biomass. Subsequently, DNeasy UltraClean Microbial Kit (Qiagen) was used to isolate DNA from the pellets according to the instructions of the manufacturer. V3 and V4 variable regions of the 16S rRNA gene were amplified with the universal primer pair SD (S-D-Bact-0341-b-S-17/S-D-Bact-0785-a-A-21) (Klindworth et al. 2013). Amplification was performed according to the 16S metagenomic sequencing library preparation guide of Illumina using KAPA HiFi HotStart Ready Mix (KAPA Biosystems). Paired-end fragment reads were generated on an Illumina MiSeq sequencer using MiSeq Reagent Kit v3 (600-cycle) by SeqOmics Biotechnology Ltd. (Mórahalom, Hungary). The raw sequencing data were analysed according to Banerjee et al. (2022) to cluster sequences into OTUs and calculate the diversity of the samples using rarefaction curves. Read numbers were between 40 000 and 50 000 during the data processing performed by MiSeq SOP of mothur v1.41.1 (Schloss et al. 2009; Kozich et al. 2013). Sequence reads were deposited in NCBI SRA under BioProject ID PRJNA704528.

**Strain isolation and Sanger-sequencing of 16S rRNA and I.2.C** ***C23O*** **genes**.

To gain bacterial isolates, 5 mL was taken from each of the 5th week enrichment cultures and mixed, then 1 mL of this pooled sample was serially diluted with 0.85% (w/v) sterile saline solution and spread on the surface of R2A plates (DSM medium No. 830) in an amount of 100 µL. After 10 days of incubation at 15 °C, bacterial colonies were picked up. The UltraClean Microbial DNA Kit (Qiagen) was used according to the instructions of the manufacturer to isolate DNA from the pure cultures made by repeated streaking. Isolated strains were identified based on the 16S rRNA gene sequence. The 16S rRNA gene of strains was amplified and sequenced using the following primers: 27F, 338F, 803F and 1492R (Soergel et al. 2012). The 16S rRNA gene sequence of the strains was determined by using BigDye Terminator version 3.1 Cycle Sequencing Kit (ThermoFisher Scientific) according to the manufacturer’s instructions. Sequencing products were separated on a Model 3130 Genetic Analyzer (Applied Biosystems). To select bacterial strains involved in microaerobic benzene degradation, PCR amplifications of the subfamily I.2.C *C23O* gene were carried out on DNA extracted from strains by using the forward primer XYLE3F, 5´-TGYTGGGAYGARTGGGAYAA-3´, and the reverse primer XYLE3R, 5´-TCASGTRTASACITCSGTRAA-3´ (Táncsics et al. 2013). Sanger sequencing of the 16S rRNA and *C23O* genes were performed according to Benedek et al. (2018).

### Phylogenetic analysis

To determine an approximate phylogenetic affiliation of strain B7^T^, the nearly complete 16S rRNA gene sequence was compared with other closely related strains available in the EzBiocloud server (https://www.ezbiocloud.net) (Yoon et al. 2017). Phylogenetic and molecular evolutionary analyses were conducted using MEGA X. (Kumar et al. 2018). Phylogenetic trees were constructed by using the neighbor-joining (NJ) (Saitou and Nei 1987), maximum-likelihood (ML) (Felsenstein 1981) and maximum-parsimony (MP) methods. Kimura’s two-parameter calculation model (Kimura 1980) was selected for NJ and ML analyses, respectively. Tree topologies and distances were evaluated by bootstrap analysis based on 1000 replicates.

### Whole genome sequencing and genome data analysis

The whole-genome sequencing of strain B7^T^ was performed by SeqOmics Biotechnology Ltd., Mórahalom, Hungary. In vitro fragment libraries were prepared using the NEBNext® Ultra™ II DNA Library Prep Kit for Illumina. Paired-end fragment reads were generated on an Illumina NextSeq sequencer using TG NextSeq® 500/550 High Output Kit v2 (300 cycles). Primary data analysis (base-calling) was carried out with Bbcl2fastq^ software (v2.17.1.14, Illumina). Trimmed sequences were *de novo* assembled and scaffolding were performed with SPAdes (v3.13.0) (Nurk et al. 2013). The genome assembly was submitted to NCBI Prokaryotic Genomes Annotation Pipeline (PGAP) v4.5 for automatic annotation (Tatusova et al. 2016). An up-to-date bacterial core gene set (UBCG, concatenated alignment of 92 core genes) and pipeline was utilized for phylogenetic tree construction as described by Na et al. (2018). Genome-to-Genome Distance Calculator (GGDC, https://ggdc.dsmz.de/) version 2.1. were used to determine the digital DNA–DNA hybridization values among strain B7^T^ and related species with available genome sequences (Meier-Kolthoff et al. 2013), which were retrieved from the GenBank database (http://www.ncbi.nlm.nih.gov/genbank). The calculations of orthologous average nucleotide identity (OrthoANI) values between strain B7^T^ and its closest relatives were performed by using the OAT software (Lee et al. 2016). The whole-genome-based phylogenetic analysis was carried out by the MiGA pipeline (http://microbial-genomes.org/) (Rodriguez et al. 2018). In order to determine which BTEX-compounds could potentially be degraded by strain B7^T^, the genome was annotated by using the Genoscope platform MAGE (Vallenet et al. 2006, 2009) and then analysis was performed by combining automated annotation from MAGE and manual curation using information from MetaCyc (Caspi et al. 2014), KEGG (Kanehisa and Goto 2000) and UniProt (Bateman 2019). CLC Genomics Workbench Tool v21 (Qiagen) was also used to identify gene clusters involved in the degradation of aromatic hydrocarbons.

### Aromatic hydrocarbon degradation analyses

To explore the ability of the strain B7^T^ to degrade BTEX components, monoaromatic hydrocarbons were added individually or in a 1:1 mixture of all BTEX components to 50 ml of MS medium supplemented with vitamins at a total concentration of 6 mg L^− 1^. The experiment was carried out in triplicates of 100-ml crimp top serum bottles under aerobic (7–8 mg L^− 1^ dissolved O_2_) and microaerobic (0.5 mg L^− 1^ dissolved O_2_) conditions. Bottles were inoculated with 100 µl suspensions of B7^T^ cells (OD_600_ 0.5) no older than 24 h. Then the bottles were incubated in a rotary shaker at 28 °C, 150 rpm Triplicates of abiotic controls were also used. Concentrations of individual or mixed BTEX compounds were detected for every 24 h by GC-MS according to the parameters mentioned above.

### Phenotypical and biochemical characteristics

Native preparation of B7^T^ was studied by Gram staining (Claus 1992) to determine the cell size, shape and arrangement. The cell morphology and flagellation type were investigated by transmission electron microscopy (H-7100; Hitachi) by applying the shadow-casting technique described by Ohad et al. (1963). Physiological and biochemical tests were performed according to the protocols of Barrow and Feltham (2004) and Smibert and Krieg (1994). The influence of temperature, salinity and pH on growth was investigated by using R2A broth based on its absorbance (600 nm). The growth test was examined between 4 and 45 °C, pH 4.0–11.0 and 0.1–7% (w/v) of NaCl. The pH of the broth was adjusted after autoclaving with sterile, 20% KOH or HCl. API tests (API ZYM, 20 NE, 50 CH test kits; bioMérieux, France) were performed according to the manufacturers’ instructions to determine the enzyme activities and other biochemical characteristics of B7^T^. Growth under anaerobic conditions was investigated in the presence of 0.15% (w/v) KNO_3_ as described earlier by Farkas et al. (2015). The whole-cell fatty acids, respiratory quinones and polar lipids were analysed by the Identification Service of DSMZ (Braunschweig, Germany). Sufficient cells of comparable physiological age could be harvested from the third streak quadrant of the R2A agar plates after cultivation at 28 °C for 48 h. Analysis of fatty acid methyl esters was performed based on the standard protocol of the Sherlock Microbial Identification System version 6.1 (MIDI; MIDI Inc., USA) and identified using the Calculation Method TSBA40 database. Identification of the summed features was performed by GC-MS analysis. The polar lipids and respiratory quinones were extracted based on the protocol modified after Bligh and Dyer (1959). The detection of the total lipid material and functional groups were performed according to described by Tindall et al. (2007). The respiratory quinones were analysed by using reversed-phase HPLC (LDC Analytical; Thermo Separation Products) using methanol:heptane 9 : 1 (v/v) as eluant.

## Results and discussion

### The composition of microaerobic benzene-degrading microbial community revealed by 16 S rRNA gene amplicon sequencing

**Fig. 1:**
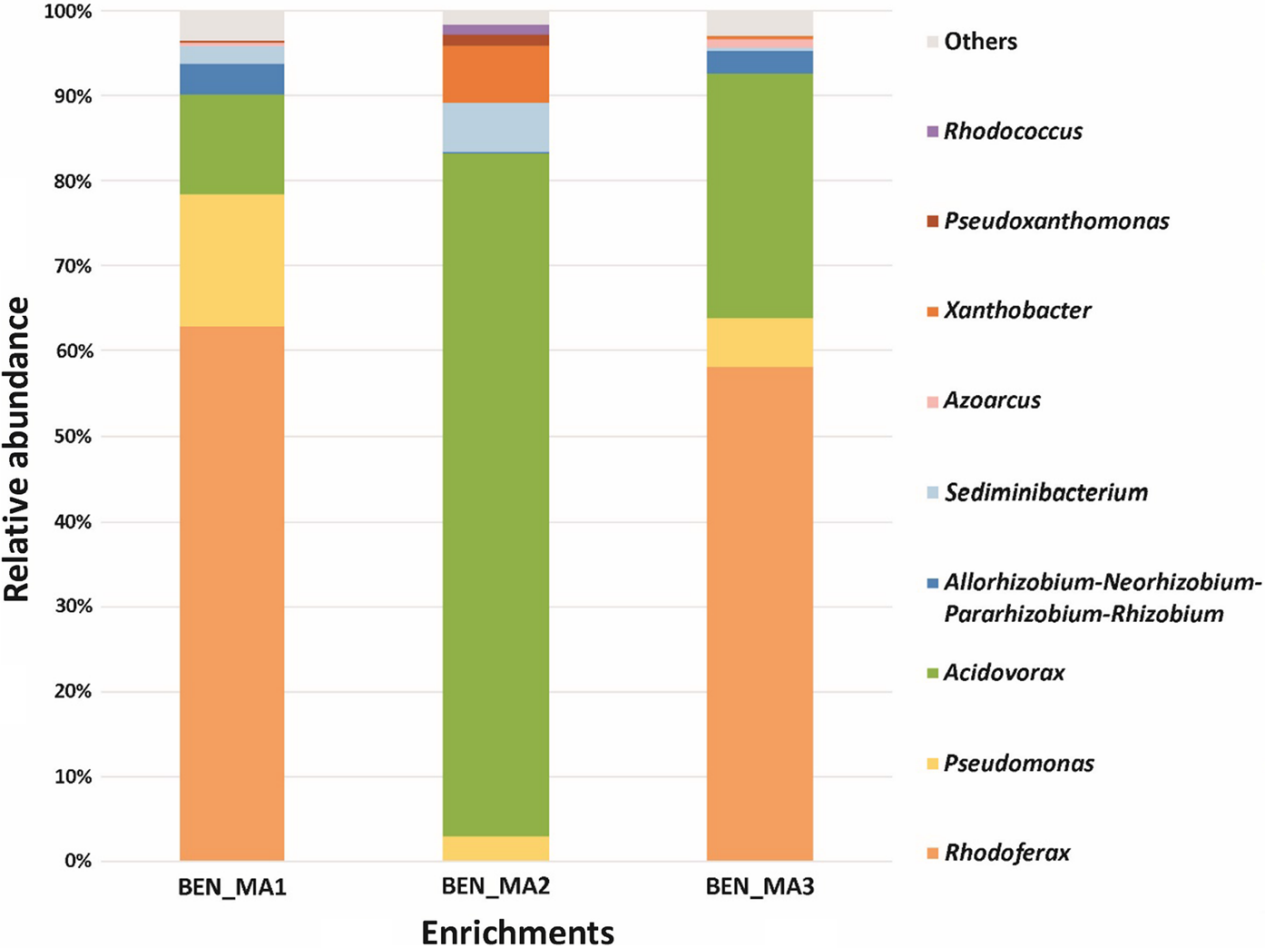
Genus-level bacterial community structure of microaerobic benzene-degrading enrichments designated BEN_MA1, BEN_MA2 and BEN_MA3 as revealed by Illumina paired-end 16S rRNA gene amplicon sequencing. Only taxa contributing more than 1% abundance were depicted

By using the BTEX-contaminated groundwater inoculum, microaerobic enrichments were set up in triplicates with benzene as a sole source of carbon and energy. Based on the measurements carried out in the 5th week of enrichment, the benzene was almost depleted by the third day of incubation from each microaerobic benzene-degrading enrichment. This result suggested that highly efficient microaerobic benzene-degrading communities evolved in the enrichments. Rarefaction curves were created for each sample (Fig. S1) and showed high sequencing coverage in all samples, suggesting that the sequencing depth was adequate. All of the samples showed OTU-based saturation around 55–65 OTUs. In two of the parallel enrichments the bacterial communities were overwhelmingly dominated by members of the genus *Rhodoferax* (BEN_MA1: 62.8%; BEN_MA3: 58.1%) followed by *Acidovorax* (BEN_MA1: 11.8%; BEN_MA3: 28.8%) and *Pseudomonas* (BEN_MA1: 15.6%; BEN_MA3: 5.6%). In case of enrichment BEN_MA2, members of the genus *Acidovorax* (79.4%) were the most dominant community members, while the abundance of *Rhodoferax* was only marginal here for some yet unknown reason (Fig. 1). Besides the most dominant genera, members of *Sediminibacterium, Xanthobacter* and *Allorhizobium-Neorhizobium-Pararhizobium-Rhizobium* were also detectable with significant abundance (> 1%) as well in at least one of the enrichment communities. Little is known about the ecological role of the genus *Rhodoferax* in hydrocarbon contaminated subsurface, as the genus contains only eight validly described species (https://lpsn.dsmz.de/genus/rhodoferax). Some previous studies have shown the presence of genus *Rhodoferax*-related bacteria in ecosystems contaminated with aromatic hydrocarbons, especially occurrence of species closely related to *Rhodoferax ferrireducens* and *R. antarticus* were common (Alfreider and Vogt 2007; Aburto and Peimbert 2011), and the role of *Rhodoferax* sp. in sulfolane degradation has also been reported (Kasanke et al. 2019). The presence of the genus *Rhodoferax* has also been previously described in the BTEX-contaminated area of Siklós (Táncsics et al. 2012, 2013; Farkas et al. 2017). The genus *Pseudomonas* contains model organisms for the studies of aerobic BTEX degradation, especially aerobic toluene degrading capability of certain *Pseudomonas* strains is deeply investigated. Aburto et al. (2011) reported the presence of all three genera (*Rhodoferax*, *Acidovorax* and *Pseudomonas*) in different samples of the SIReN site (UK) and suggested that these bacteria were potential benzene and toluene degraders even under microaerobic conditions. *Acidovorax* genus related bacteria are frequently observed in petroleum hydrocarbon–contaminated environments (Singleton et al. 2018; Révész et al. 2020a,b), e.g., bacteria most closely related to *Acidovorax defluvii* and *A. delafieldii* were detected in BTEX-contaminated environments (Alfreider and Vogt 2007; Révész et al. 2020a, b). According to previous studies, members of the genera *Acidovorax* and *Pseudomonas* occurred in benzene-, toluene-, and xylene-degrading enrichments as well (Révész et al. 2020a; Banerjee et al. 2022). The genus *Sediminibacterium* and *Allorhizobium-Neorhizobium-Pararhizobium-Rhizobium* have been also found to be present in petroleum-contaminated environments (Lindstrom et al. 2003; Yessica et al. 2013; Poi et al. 2018; Banerjee et al. 2022; Chen et al. 2022). Members of the genus *Xanthobacter* are often used for biodegradation purposes, as they can play role in the degradation of chlorinated hydrocarbons, alkenes, cyclohexane and phenol as well (van Ginkel and de Bont 1986; Janssen et al. 1989;; Trower et al. 1985; Nagamani et al. 2009).

### Isolates

Based on different colony morphology and growth pattern altogether 13 strains were isolated from the pooled sample of the enrichments, which represented eight different genera (Table S1): *Brucella, Pseudomonas, Rhizobium, Rhodococcus, Ideonella, Xanthobacter, Pseudoxanthomonas* and *Pinisolibacter*. Most of the isolates belonged to the *Hyphomicrobiales* (*Hyphomicrobiales* -synonym: *Rhizobiales*), many of which are known to occur in ecosystems contaminated with petroleum hydrocarbons and some of them are potential hydrocarbon degraders (Rochman et al. 2017). Several members of the genus *Rhodococcus, Pseudoxanthomonas* and *Pseudomonas* are also known to degrade a wide range of xenobiotics (Táncsics et al. 2008; Wasi et al. 2013; Rochman et al. 2017; Lu et al. 2019; Révész et al. 2020b). Some members of these genera involved in petroleum hydrocarbon degradation, e.g., *Rhodococcus aetherivorans* was isolated from a mixture of petroleum compounds (Auffret et al. 2009) and the ability of members of the genus *Pseudoxanthomonas* to metabolize aromatic hydrocarbons and other pollutants are also studied (Patel et al. 2012; Kim 2008). The 16S rRNA gene sequences of the isolates were deposited at GenBank under the accession numbers OM570587-OM570577, MZ041034 and MZ047316 subsequently. The lack of *Acidovorax* and *Rhodoferax* isolates is striking, however it is a well known phenomenon that culture dependent, and independent microbial ecological approaches tend to yield results with minimal overlap.

Amid the isolates, only the *Ideonella* strain designated as B7 possessed subfamily I.2.C-type *C23O* gene. The closest relatives of this *C23O* gene were found in other species of the genus *Ideonella*, for instance in *Ideonella dechloratans* CCUG 30977^T^ and in *Ideonella* sp. strain B508-1 with a low sequence similarity (98.41% and 98.09%) suggesting that occurrence of the *C23O* gene is not unique among the members of the genus. Genus *Ideonella* was also detectable by 16 S rRNA gene amplicon sequencing in the enrichment communities, although with low abundance (< 1%). These results suggest that the strain *Ideonella* sp. B7 was a relevant member of the microaerobic benzene-degrading enrichment cultures involved actively in BTEX degradation. Consequently, strain B7 was chosen for further investigation to explore its role in microaerobic benzene-degradation and to clarify its phylogenetic position.

### Phylogenetic analysis of strain B7^T^

**Fig. 2:**
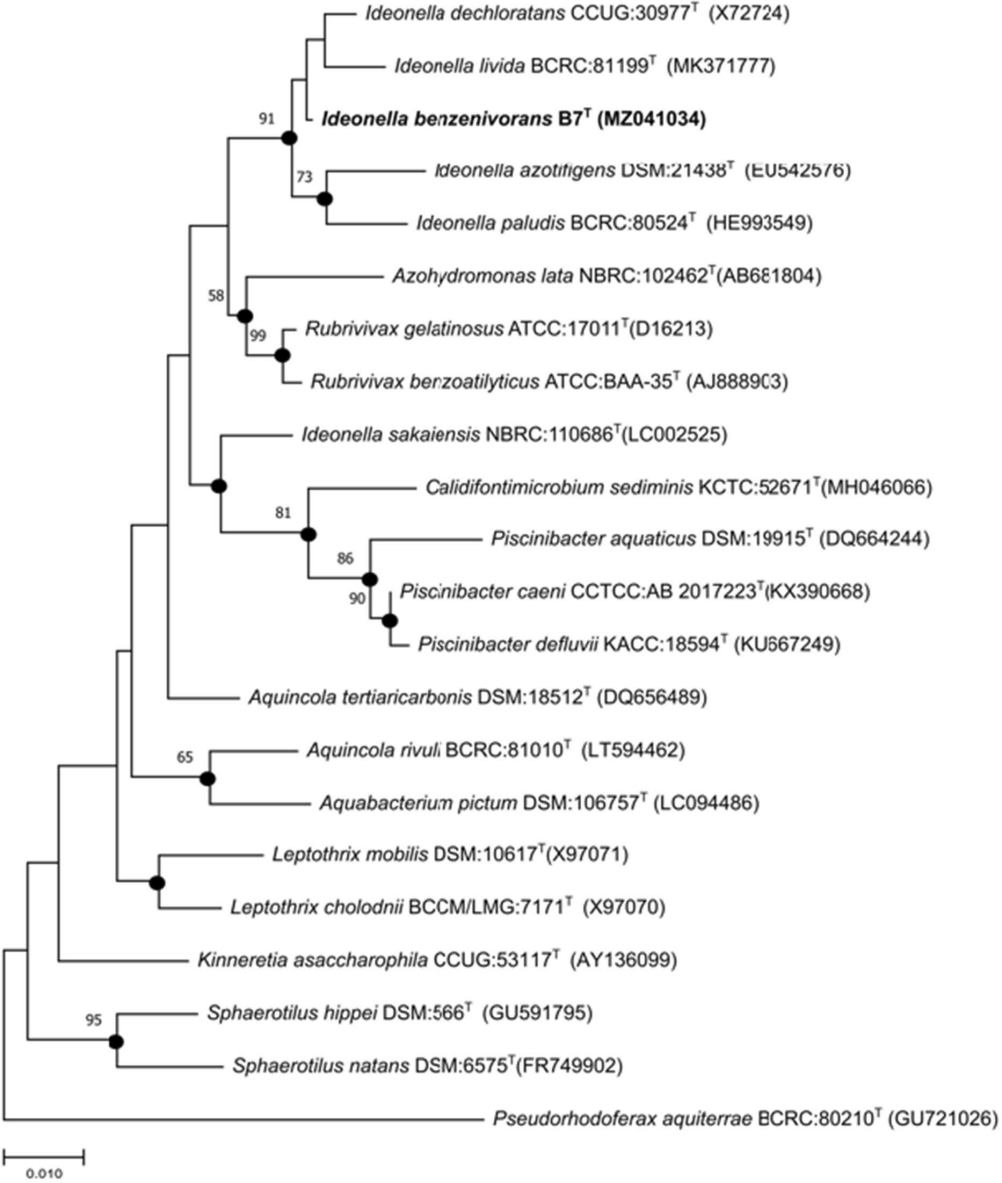
Maximum-likelihood phylogenetic tree with Kimura’s two-parameter calculation model based on 16S rRNA gene sequences highlighting the position of *Ideonella benzenivorans* strain B7^T^ relative to other closely related species. Filled circles indicate that the corresponding nodes were also recovered using the maximum-likelihood (ML), neighbour-joining (NG) and maximum-parsimony (MP) tree-making algorithms. Sequences were aligned using CLUSTAL W with default parameters and phylogenies were carried out by the software MEGA X. Bootstrap values (> 50%) based on 1000 bootstrap replicates are shown at branch nodes. *Pseudorhodoferax aquiterrae* BCRC:80210^T^ was used as an out-group. The respective GenBank accession numbers for 16S rRNA genes are indicated in parentheses. Bar, 0.01 substitutions per nucleotide site

The 16 S rRNA gene seqence of strain B7^T^ was 1413 bp long and it was deposited at GenBank database under the accession number MZ041034. Phylogenetic analysis based on the 16 S rRNA gene sequence showed that strain B7^T^ formed a lineage within the family *Comamonadaceae* and clustered as a member of the genus *Ideonella* (Fig. 2). Comparative 16 S rRNA gene sequence analysis revealed that strain B7^T^ was most closely related to *Ideonella dechloratans* CCUG 30977^T^ (99.15%) followed by *I. livida* TBM-1^T^ (98.51%), *I. azotifigens* 1a22^T^ (98.28%), *I. paludis* KBP-31^T^ (98.08%) and *I. sakaiensis* 201-F6^T^ (98.02%). Strain B7^T^ clustered with *I. dechloratans*, and *I. livida* in all of the three (NJ, ML and MP) phylogenetic trees. Furthermore, it formed a separate lineage within this cluster in ML and MP trees, which fact supported the identification of strain B7^T^ as a possible novel member of the genus *Ideonella* (Figs. 2, S2, S3).

### Genomic analysis of strain B7^T^

**Fig. 3:**
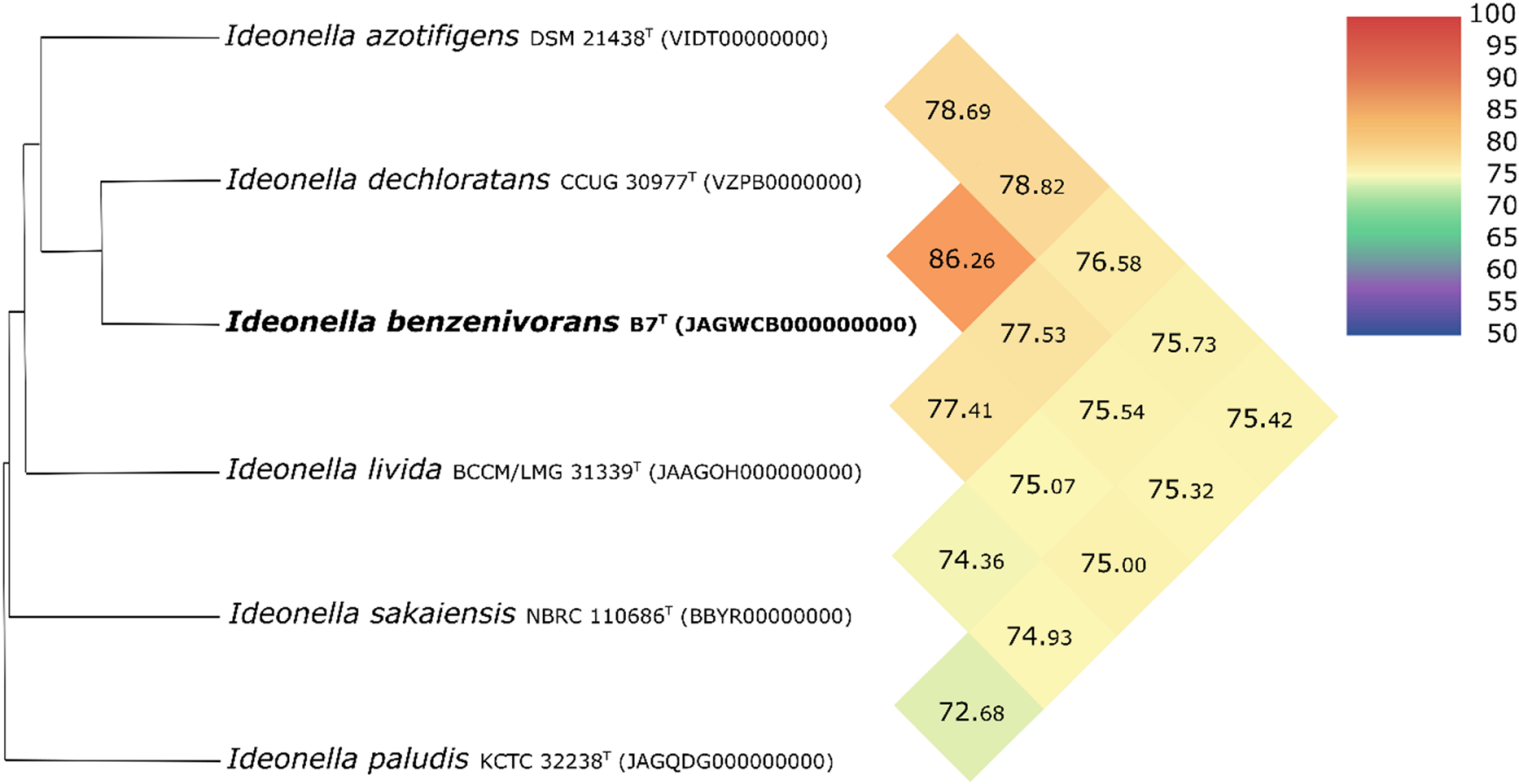
Heatmap generated with OrthoANI values calculated using the OAT software between strain B7^T^ and other closely related *Ideonella* species. The colour code indicates the closest species in red to the farthest in green

**Fig. 4:**
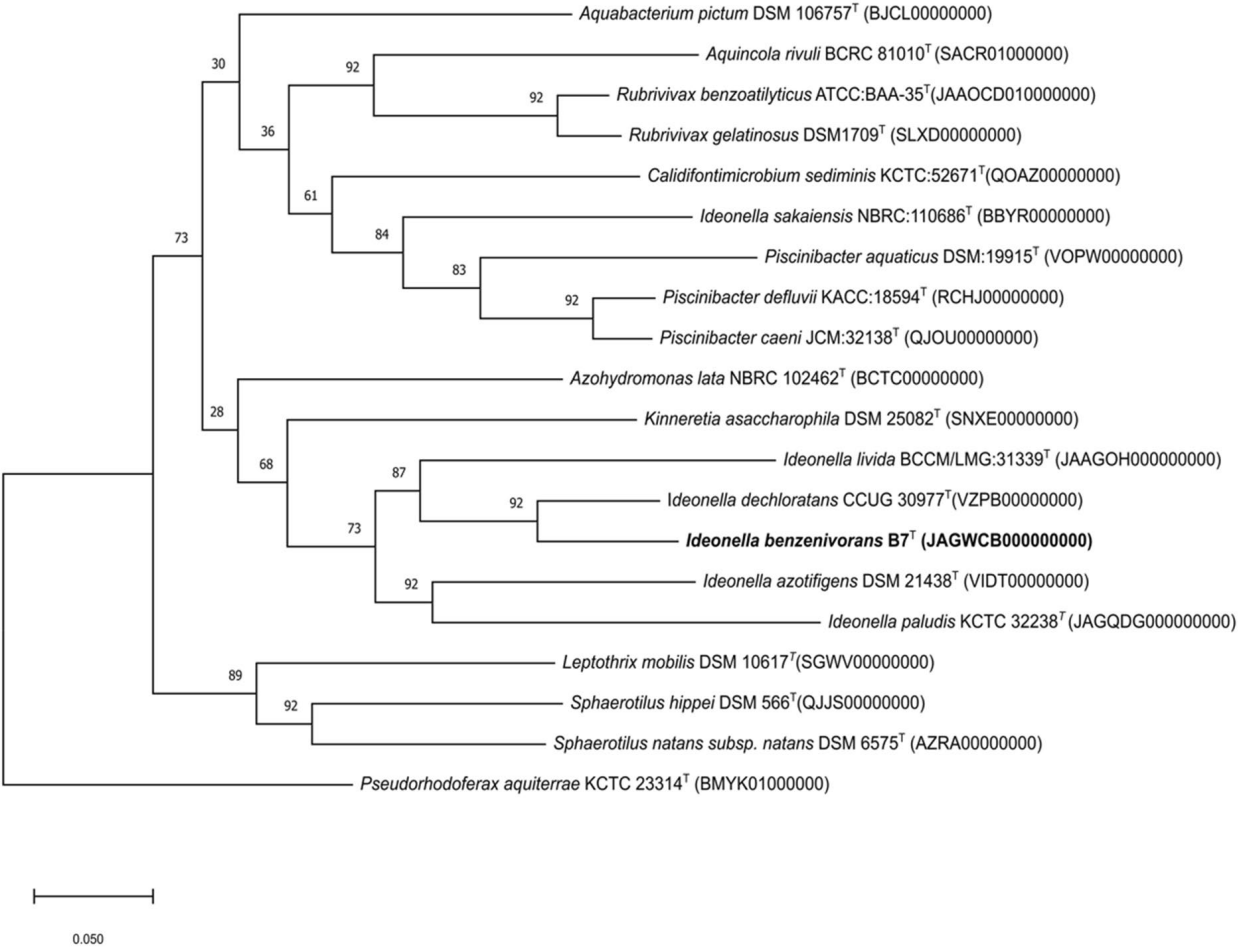
Phylogenomic tree constructed using UBCGs (concatenated alignment of 92 core genes). Bar, 0.05 substitution per nucleotide position

Whole genome sequence of strain B7^T^ has been deposited at GenBank under the accession number JAGWCB000000000. The high-quality draft genome of strain B7^T^ had a total length of 4,490, 200 bps. The genome sequence was assembled into 21 contigs and has an average coverage value of 1823. The DNA G + C content of strain B7^T^ is 68.8%, which value falls within the range of *Ideonella* species. The MiGA pipeline indicated high completeness value for the whole-genome sequence (100%), while contamination was low (0.9%). The number of predicted proteins was 4044. The dDDH values were much lower than the species threshold of 70% recommended for species demarcation (Chun et al. 2018). dDDH relatedness between strain B7^T^ and *Ideonella dechloratans, Ideonella azotifigens* and *Ideonella livida* were 30.2%, 22.7% and 22.0%, respectively. In case of the other *Ideonella* species, the dDDH value was below 22%. Moreover, OrthoANI values were also below the treshold values (95–96%) recommended for the species level delineation (Chun et al. 2018) (Fig. 3). These results indicated that strain B7^T^ should be assigned to a novel species of the genus *Ideonella*. Furthermore, a genome-based phylogenetic tree was also reconstructed with the UBCGs software (Fig. 4). The phylogenetic tree based on the coding sequences of 92 protein clusters showed that strain B7^T^ formed a distinct phyletic lineage of the genus *Ideonella*.

**Fig. 5:**
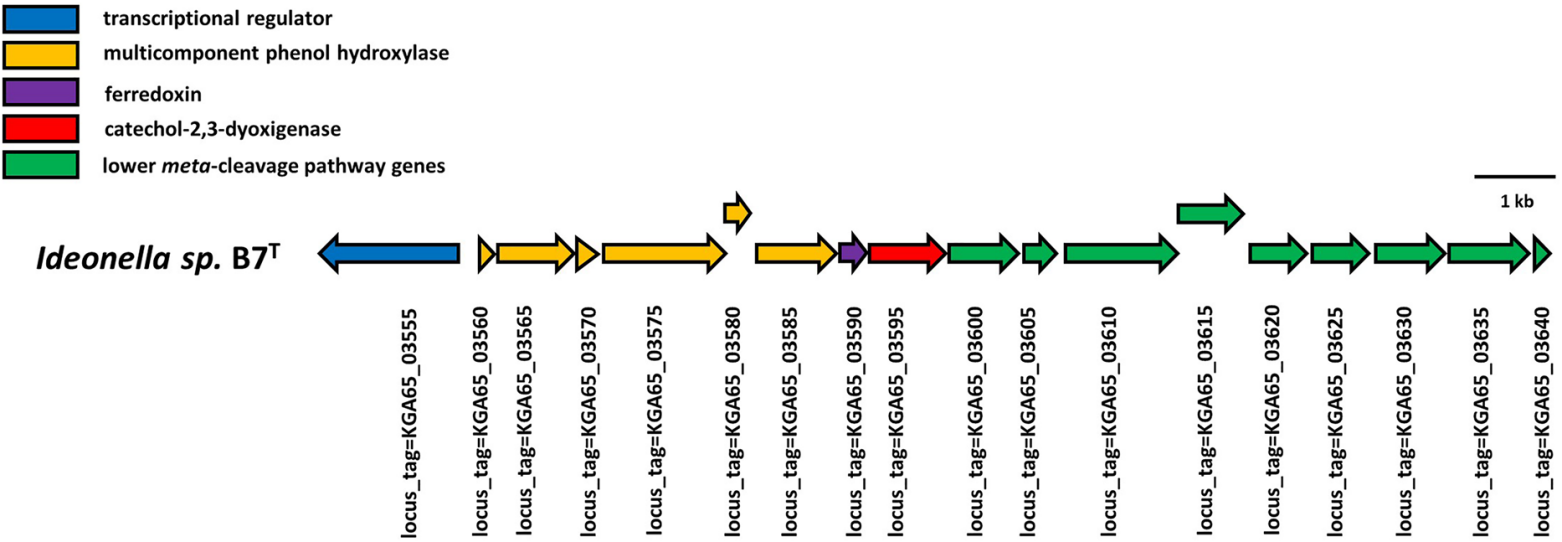
Physical map of the phenol degradation gene cluster encoding the multicomponent phenol hydroxylase (mPH) components, the catechol 2,3-dioxygenase and other lower *meta*-cleavage pathway enzymes in the genome of strain B7^T^. The map was performed using CLC Genomics Workbench Tool version 21. Arrows represent predicted ORFs, the direction of the arrow represents the direction of transcription. The colours denote different functional groups of genes involved in aromatic hydrocarbon degradation

The catechol 2,3-dioxygenase (*C23O*) gene was found in a phenol degradation gene cluster, encoding a multicomponent phenol hydroxylase (mPH) together with a complete *meta*-cleavage pathway (Fig. 5), providing aromatic hydrocarbon-degrading ability to strain B7^T^. The operon is regulated by a σ54-interacting transcriptional regulator and it contains Rieske-type ferredoxin that mediates electron transfer from the reductase to the oxygenase. Benzene, toluene and ethylbenzene degradation pathways were annotated by using the Genoscope platform MAGE, predicting which compounds are degradable by B7^T^. For the strain B7^T^, the target *C23O* gene (about 800 bp) was also detected successfully with Sanger-sequencing. The I.2.C catechol-2,3-dioxygenase gene sequence of strain B7^T^ obtained by Sanger-method was compared with the *C23O* gene sequence extracted from the genome assembly and showed 100% similarity.

The presence of a subfamily I.2.C-type *C23O* gene in the genome implied the possibility that strain B7^T^ is capable of degrading monoaromatic componds under microaerobic conditions. Analyses of the genome of strain B7^T^ revealed the presence of genes encoding *cbb3*-type cytochrome *c* oxidase (between locus tags KGA65_1305 and KGA65_13040), further hinting on this possibility. It is well known that this oxidase has high affinity for oxygen and is expressed under microaerobic conditions (van der Oost et al. 1994). Besides, genes encoding cytochrome *o* ubiquinol oxidase were also detected in the genome (locus tags KGA65_11435–KGA65_11455), assuming that under ample supply of oxygen this oxidase accommodates most of the electron flow (Dinamarca et al. 2002).

### Aromatic hydrocarbon degradation analyses

**Fig. 6:**
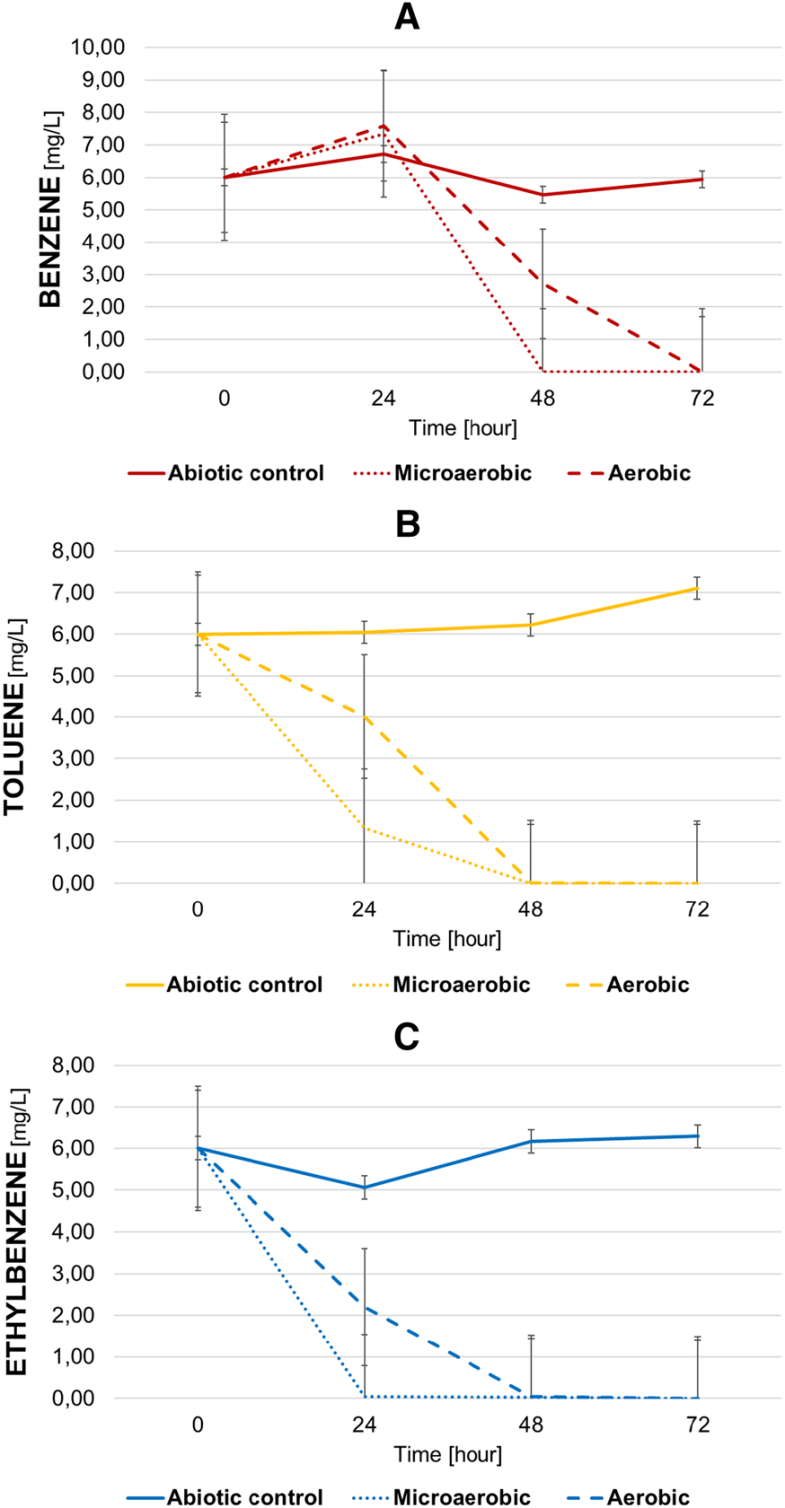
Aerobic and microaerobic degradation of **a** benzene, **b** toluene, **c** ethylbenzene by *Ideonella* sp. strain B7^T^. Abiotic controls were used for each experiment. The concentration of BTEXs were measured by GC-MS analysis for every 24 h. The averages of triplicate experiments ± standard errors of the means, indicated by error bars, are shown

As predicted by the genome analysis, it was observed that strain B7^T^ was capable of degrading both aerobically and microaerobically benzene, toluene and ethylbenzene as sole source of carbon and energy (Fig. 6). The aerobic degradation of toluene and ethylbenzene was completed within 48 h of incubation at 28 °C and degradation of same concentration of benzene within around 72 h under similar conditions. However, microaerobic degradation proved to be slightly more efficient. Considerable degradation of toluene and ethylbenzene was observed within 24 h and the total amount of benzene was utilized by strain B7^T^ within approximetaly 48 h. Strain B7^T^ was unable to utilize xylenes as a sole carbon and energy source. In contrast, in those experimental set-ups where the mixture of all BTEX components were present at the same time, the amount of all xylene-isomers were also significantly reduced within 48 h indicating co-metabolism of xylenes in the presence of other monoaromatic hydrocarbons (data not shown).

### Phenotypical and biochemical characteristics

*Ideonella dechloratans* CCUG 30898^T^ and *I. azotifigens* 1a22^T^ DSM 21438^T^ were used as reference strains for the comparative phenotypic analyses. Strain B7^T^ proved to be strictly aerobic, as growth was not observed in anaerobic R2A broth supplemented with KNO_3_. The cells of strain B7^T^ are rod-shaped, Gram-stain-negative and motile with polar flagellum. The approximate lenght of the cells was 1.8–2.0 μm (Fig. S4). The small and medium size colonies are pale amber, entire round, shiny and peak shaped after 2–3 days of incubation on R2A agar at 28 °C. Strain B7^T^ hydrolysed Tweens 80, urea, esculin and gelatin, showed positive results for oxidase and casease and weakly positive for catalase. Strain B7^T^ grew well but the reference strains were unable to grow at 45 °C. In addition, intense growth was observed after 24 h incubation at 35 °C on R2A plates. In API ZYM tests, B7^T^ showed high diversity of enzymatic activities similar to the reference strains, but based on API 20NE and 50CH tests, strain B7^T^ was unable for the assimilation and fermentation of any carbohydrates of those that were examined. Other differential physiological characteristics of B7^T^ are given in Table 1 with its closest relatives.

**Table 1:** Major phenotypic and biochemical characteristics of 1, strain B7^T^; 2, *Ideonella dechloratans* CCUG 30898^T^; 3, *Ideonella azotifigens* DSM 21438^T^; +, positive; -, negative; W, weakly positive. All data are from the present study except those marked with *

Characteristic	1	2	3
Colony colour	pale amber	white	white
Temperature range (Celsius)	15–45	12–42*	4–35*
pH range	4–10	5–9*	6–8*
NaCl tolerance (% w/v)	2	3>*	0.5>*
Catalase	w	+	+
Phosphatase	−	w	+
H_2_S production	−	+	−
**Hydrolysis of**			
Starch	−	+	−
**Assimilation of (API 20NE)**			
D-glucose	−	+	−
**Enzyme activities (API ZYM)**			
Alkaline phosphatase	w	−	+
Lipase (C14)	w	−	−
Cystine arylamidase	−	w	w
Trypsin	w	−	−
Acid phosphatase	−	w	+
α-glucosidase	+	+	w
N-acetyl- β-glucosaminidase	w	−	−
**Acid production from (API 50CH)**			
Glycerol	−	−	+
D-arabinose	−	−	+
L-arabinose	−	−	+
D-ribose	−	−	+
D-galactose	−	−	+
D-glucose	−	−	+
D-fructose	−	−	+
D-mannose	−	−	+
L-rhamonose	−	−	+
Inositol	−	−	+
D-mannitol	−	−	+
D-sorbitol	−	−	+
Esculin ferric citrate	−	−	+
D-cellobiose	−	−	+
D-maltose	−	−	+
D-fucose	−	−	+
L-fucose	−	−	+

The major fatty acids of strain B7^T^ were C_16:0_ and summed feature 3 (C_16:1_ *ω*7c/iso-C_15:0_ 2-OH) and C_18:1_ *ω7*c was present in significant quantities, similar with the reference strains. The additional GC-MS analysis revealed that summed feature 3 contained only the fatty acid C_16:1_ *ω*7c. The presence of C_15:0_ and C_16:1_ 2-OH differentiates strain B7^T^ from other phylogenetically related species of the genus *Ideonella* (Table 2). The sole respiratory quinone was ubiquinone-8 (Q-8) and the major polar lipids were phosphatidylethanolamine (PE), diphosphatidylglycerol (DPG) and phosphatidylglycerol (PG). In addition, an unidentified aminophospholipid (APL) an aminolipid (AL) and a polar lipid (L) were also detected (Fig. S5).

**Table 2:** Cellular fatty acid compositions of strain B7^T^ and related species. Taxa: 1, strain B7^T^; 2, *Ideonella dechloratans* CCUG 30898^T^; 3, *Ideonella azotifigens* DSM 21438^T^; Data are expressed as percentages of total fatty acids. -, Not detected. Fatty acids which were lower than 1.0% in all strains are not shown. All data are from the present study

Fatty acid	1	2	3
**Saturated**			
C_12:0_	0.7	1.1	8.0
C_14:0_	2.6	1.7	1.2
C_15:0_	1.4	−	−
C_16:0_	28.9	31.5	26.5
**Unsaturated**			
C_18:1_ *ω7*c	7.7	10.3	13.0
**Hydroxy**			
C_10:0_ 3-OH	2. 7	2.6	2.9
C_12:0_ 2-OH	1.9	2.3	−
C_12:0_ 3-OH	4.2	3.7	4.6
C_14:0_ 2-OH	5.1	2.5	−
C_16:1_ 2-OH	3.1	−	−
Cyclic			
C_17:0_ cyclo	2.6	1.5	3.7
Summed feature*			
3	36.5	38.3	38.8

*Summed features represent groups of two or three fatty acids that could not be separated by gas–liquid chromatography with the MIDI system. Summed features: 3, C_16:1_ *ω*7c/iso-C_15:0_ 2-OH. For identity confirmation and to resolve summed features of the MIDI analysis, the analysis was supplemented by a GC-MS run that resulted C_16:1_ *ω*7c

Based on above discussed data, strain B7^T^ represents a novel species within the genus *Ideonella* for which the name *Ideonella benzenivorans* sp. nov. is proposed.

**Description of** ***Ideonella benzenivorans*** **sp. nov.**

*Ideonella benzenivorans* (ben.ze.ni.vo’rans. N.L. n. *benzenum* benzene; L. v. *vorare* to devour; L. part. adj. *vorans* devouring, digesting; N.L. part. adj. *benzenivorans* digesting benzene).

The cells (1.8–2.0 μm long and 0.6–0.7 μm wide) are rod-shaped, strictly aerobic, Gram-stain-negative and motile with a polar flagellum. Colonies on R2A are small and medium size, pale amber, circular, shiny and peak shaped after 2–3 days at 28 °C. Colonies grow at 15–45 °C (optimum, 28–37 °C) and pH 4.0–10.0 (optimum pH, 6.0–8.0) and can tolerate maximum 2% NaCl (w/v) (optimum 0.3% w/v). Oxidase and casease are positive and catalase is weakly positive. The type strain is able to reduce nitrate to nitrite or ammonium and can perform hydrolysis of Tween 80, urea, esculin and gelatin. Glucose and other carbohydrates are not metabolised. The type strain shows the following enzyme activities: positive for esterase (C4), esterase lipase (C8), leucine arylamidase, valine arylamidase, *α*-chymotrypsin, napthol-AS-BI-phosphohydrolase, *α*-glucosidase, weakly positive for alkaline phosphatase, lipase (C14), trypsin, *N*-acetyl-*β*-glucosaminidase. The sole respiratory quinone is Q-8. The major polar lipids are phosphatidylethanolamine, diphosphatidylglycerol and phosphatidylglycerol. The major fatty acids are C_16:0_ and C_16:1_ *ω*7c. The DNA G + C content of the type strain is 68.8%. It is able to degrade aerobically and microaerobically benzene, toluene ethylbenzene as sole source of carbon and energy, and the xylene isomers are degraded co-metabolically in the presence of the other BTEX components.

The type strain B7^T^ (= LMG 32345^T^ = NCAIM B.02664^T^) was isolated from a benzene-degrading enrichment culture inoculated with groundwater sample of the „Siklós” BTEX contaminated site (Hungary). The GenBank accession numbers for the 16 S rRNA gene sequence and the whole genome sequence of strain B7^T^ are MZ041034 and JAGWCB000000000, respectively.

## Supplementary information

Below is the link to the electronic supplementary material.

Supplementary Material 1 (PDF 1270KB)

## Data Availability

Correspondence and requests for materials should be addressed to A.T. The whole genome data of *Ideonella benzenivorans* B7^T^ could be accessed under accession number JAGWCB000000000 in the NCBI database (www.ncbi.nlm.nih.gov). The 16 S rRNA gene amplicon sequence reads were deposited in NCBI SRA under BioProject ID PRJNA704528. The 16 S rRNA gene sequences of the isolates were deposited at NCBI GenBank under accession numbers OM570587-OM570577, MZ041034 and MZ047316.
