# Supplementary material for: Microaerobic enrichment of benzene-degrading bacteria and description of Ideonella benzenivorans sp. nov., capable of degrading benzene, toluene and ethylbenzene under microaerobic conditions
Source: Antonie Van Leeuwenhoek. 2022 Jul 16;115(9):1113–28. doi: 10.1007/s10482-022-01759-z (PMC9363352; doi:10.1007/s10482-022-01759-z)
Supplement: Supplementary file 1 — Supplementary Material 1 (PDF 1270KB) [file 10482_2022_1759_MOESM1_ESM.pdf]

Microaerobic enrichment of benzene-degrading bacteria and description of *Ideonella benzenivorans* sp. nov., capable of degrading benzene, toluene and ethylbenzene under microaerobic conditions

Anna Bedics[1], András Táncsics[1,*] Erika Tóth[2], Sinchan Banerjee[1], Péter Harkai[3], Balázs Kovács[1], Károly Bóka[4], Balázs Kriszt[3]

[1]Department of Molecular Ecology, Institute of Aquaculture and Environmental Safety, Hungarian University of Agriculture and Life Sciences, Gödöllő, Hungary;
[2]Department of Microbiology, Eötvös Loránd University, Budapest, Hungary;
[3]Department of Environmental Safety, Institute of Aquaculture and Environmental Safety, Hungarian University of Agriculture and Life Sciences, Gödöllő, Hungary
[4]Department of Plant Anatomy, Eötvös Loránd University, Budapest, Hungary;

*Correspondence: András Táncsics, Department of Molecular Ecology, Institute of Aquaculture and Environmental Safety, Hungarian University of Agriculture and Life Sciences, Páter K. u. 1., H-2100 Gödöllő, Hungary. E-mail: tancsics.andras@uni-mate.hu

Supplementary material for publication in Antonie van Leeuwenhoek online.

Table S1. Identity of bacterial strains isolated from the benzene-degrading enrichments

Strain No.	GenBank accession numbers	Closest relative (type strain)	Length of 16S rDNA analysed (bp)	Similarity (%)	Subfamily I.2.C *C23O* gene
**B1**	OM570587	*Brucella cytisi* ESC1^{T} (AY776289)	1367	100	-
**B2**	OM570586	*Pseudomonas extremaustralis* 14-3^{T} (AHIP01000073)<br><br>*Pseudomonas fildesensis* KG01^{T} (MK859934)	1360	99,78	-
**B3**	OM570585	*Rhizobium daejeonense* KCTC 12121^{T} (AY341343)	1298	98,15	-
**B4**	OM570584	*Rhodococcus aetherivorans* 10bc312^{T} (AF447391)	1417	99,85	-
**B5**	OM570583	*Pseudomonas moorei* RW10^{T} (AM293566)	1438	99,86	-
**B6**	OM570582	*Brucella cytisi* ESC1^{T} (AY776289)	1363	100	-
**B7**	MZ041034	*Ideonella dechloratans* CCUG 30898^{T} (X72724)	1413	99,15	+
**B8**	OM570581	*Rhodococcus aetherivorans* 10bc312^{T} (AF447391)	1405	99,93	-
**B9**	OM570580	*Pseudomonas fildesensis* KG01^{T} (MK859934)	1414	99,36	-
**B10**	OM570579	*Xanthobacter flavus* 301^{T} (X94199)	1386	99,78	-
**B11**	OM570578	*Pseudoxanthomonas humi* THG-MM13^{T} (KM598260)	1430	98,51	-
**B12**	OM570577	*Rhizobium selenitireducens* ATCC BAA-1503^{T} (JAEG01000027)	1367	99,78	-
**B13**	MZ047316	*Pinisolibacter ravus* E9^{T} (KY087994)	1366	97,36	-

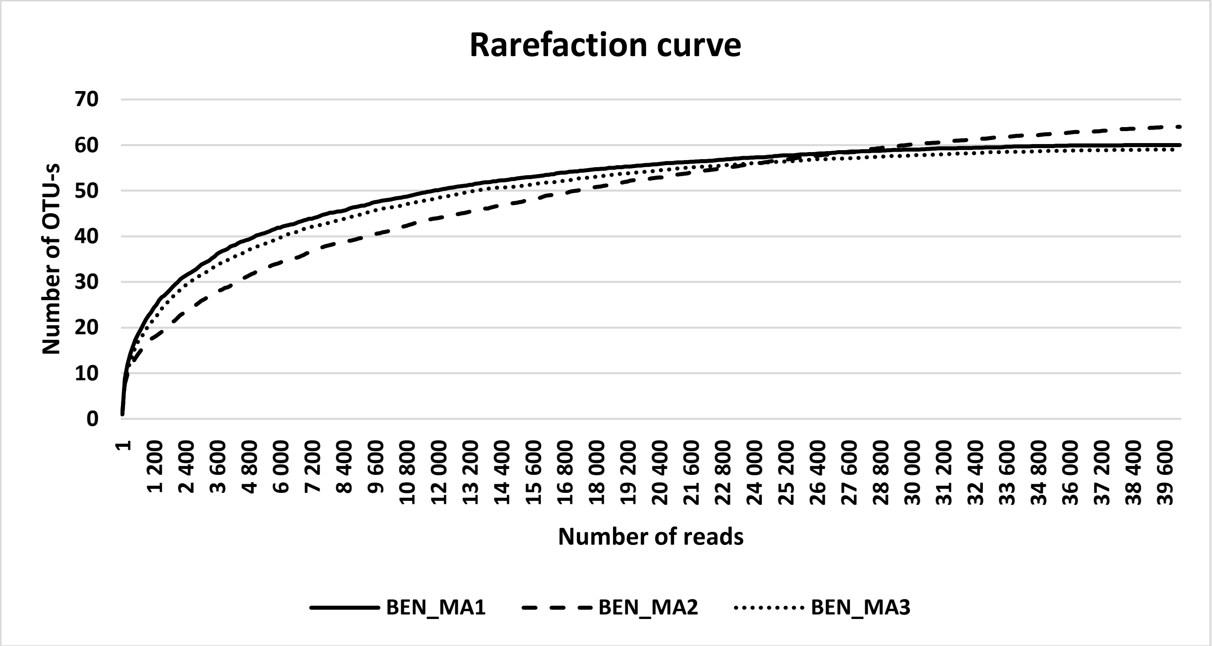

Figure S1. Rarefaction curves of the 16S rDNA amplicon sequencing from microaerobic benzene-degrading enrichments designated BEN1_MA1, BEN2_MA2 and BEN3_MA3. Rarefaction analysis was conducted using mothur v1.41.1 suggesting that the sequencing depth was adequate. The number of reads and corresponding OTUs were used to construct rarefaction curves by random sampling of all reads.

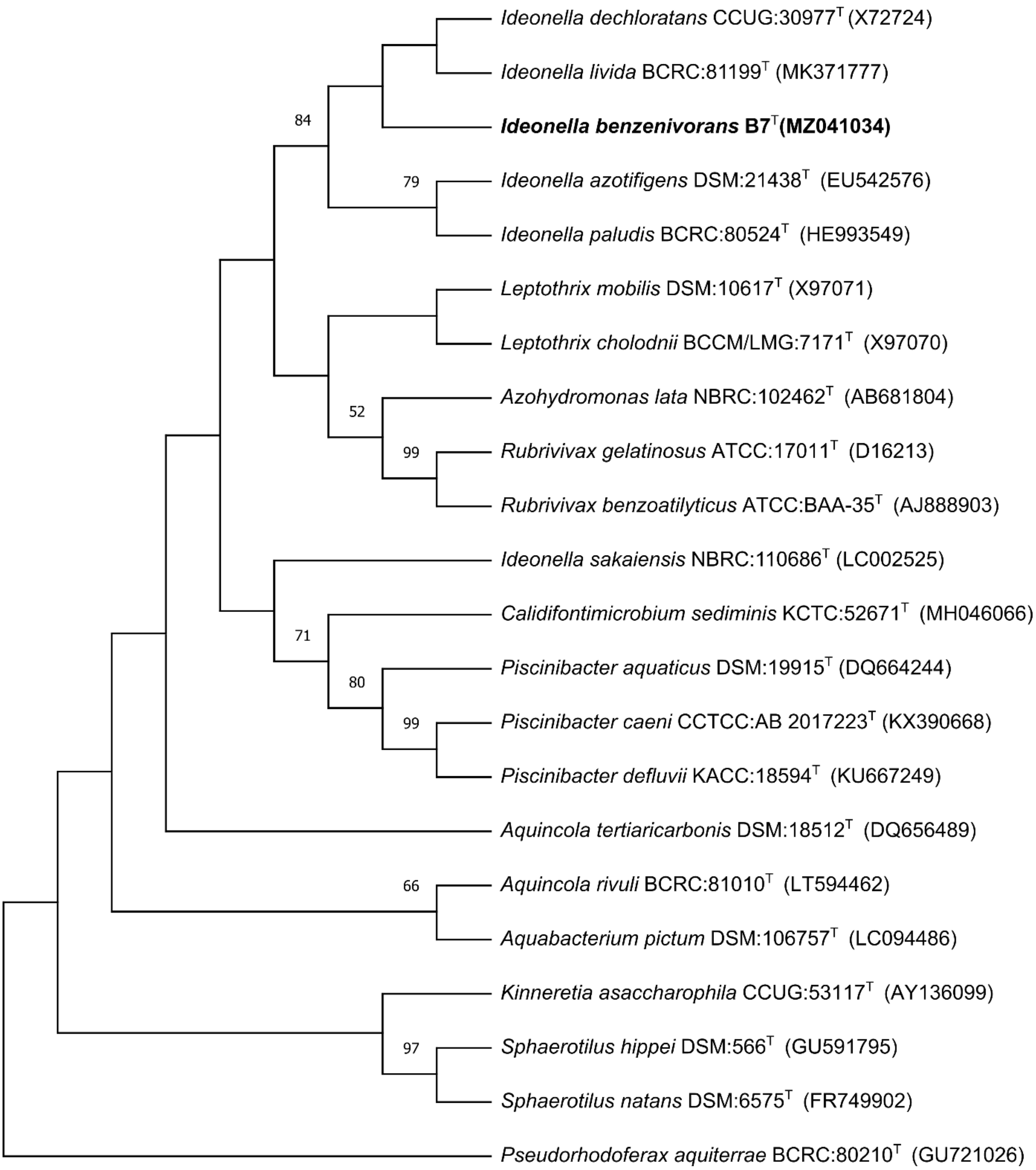

Figure S2. Maximum-parsimony phylogenetic tree based on 16S rRNA gene sequences highlighting the position of *Ideonella benzenivorans* strain B7^{T} relative to other closely related species. Sequences were aligned using CLUSTAL W with default parameters and phylogenies were carried out by the software MEGA X. Bootstrap values (>50%) based on 1000 bootstrap replicates are shown at branch nodes. *Pseudorhodoferax aqiterrae* BCRC:80210^{T} was used as an out-group.

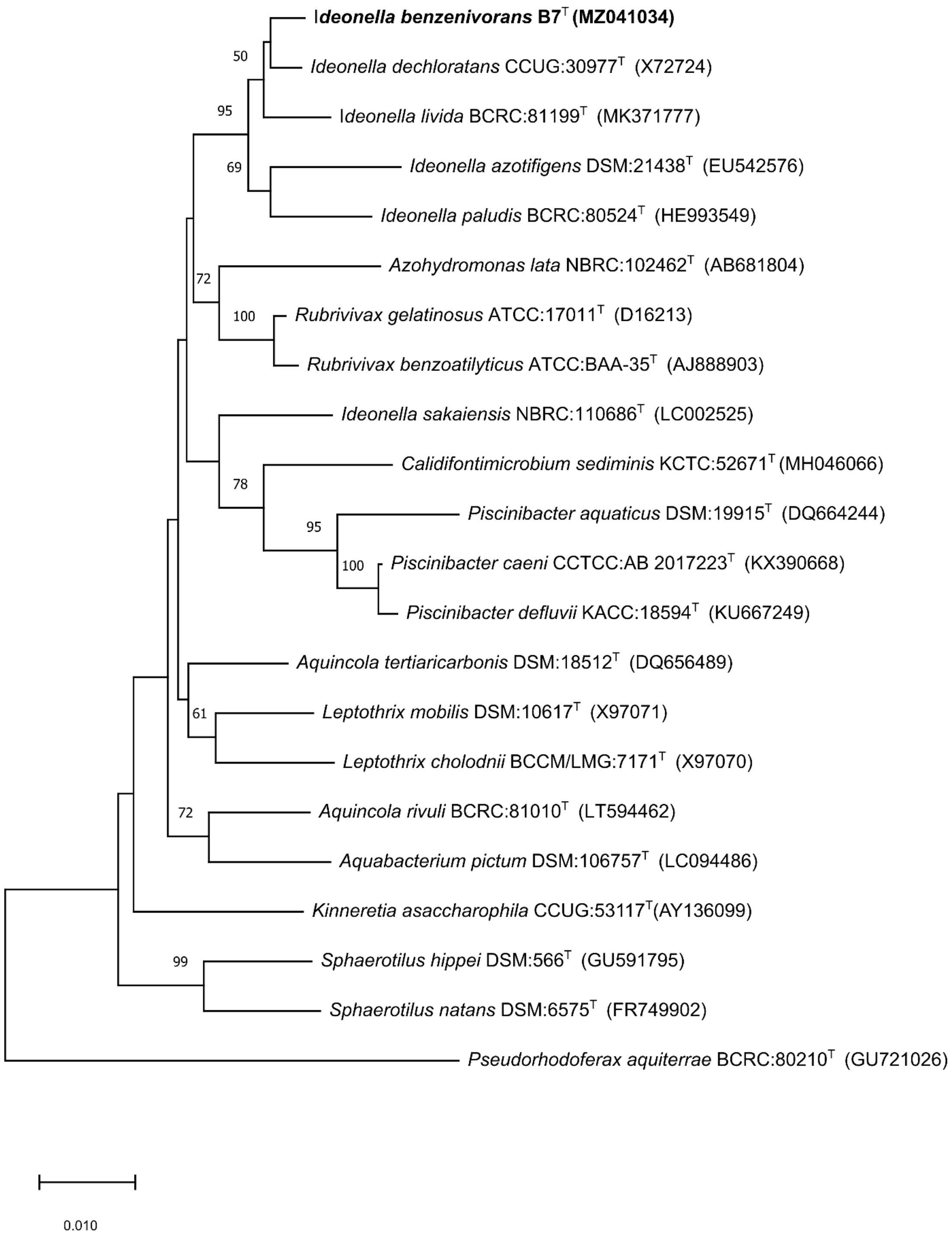

Figure S3. Neighbor-joining phylogenetic tree with Kimura's two-parameter calculation model based on 16S rRNA gene sequences highlighting the position of *Ideonella benzenivorans* strain B7^{T} relative to other closely related species Sequences were aligned using CLUSTAL W with default parameters and phylogenies were carried out by the software MEGA X. Bootstrap values (≥50%) based on 1000 bootstrap replicates are shown at branch nodes. *Pseudorhodoferax aqiterrae* BCRC:80210^{T} was used as an out-group. Bar, 0.010 substitutions per site.

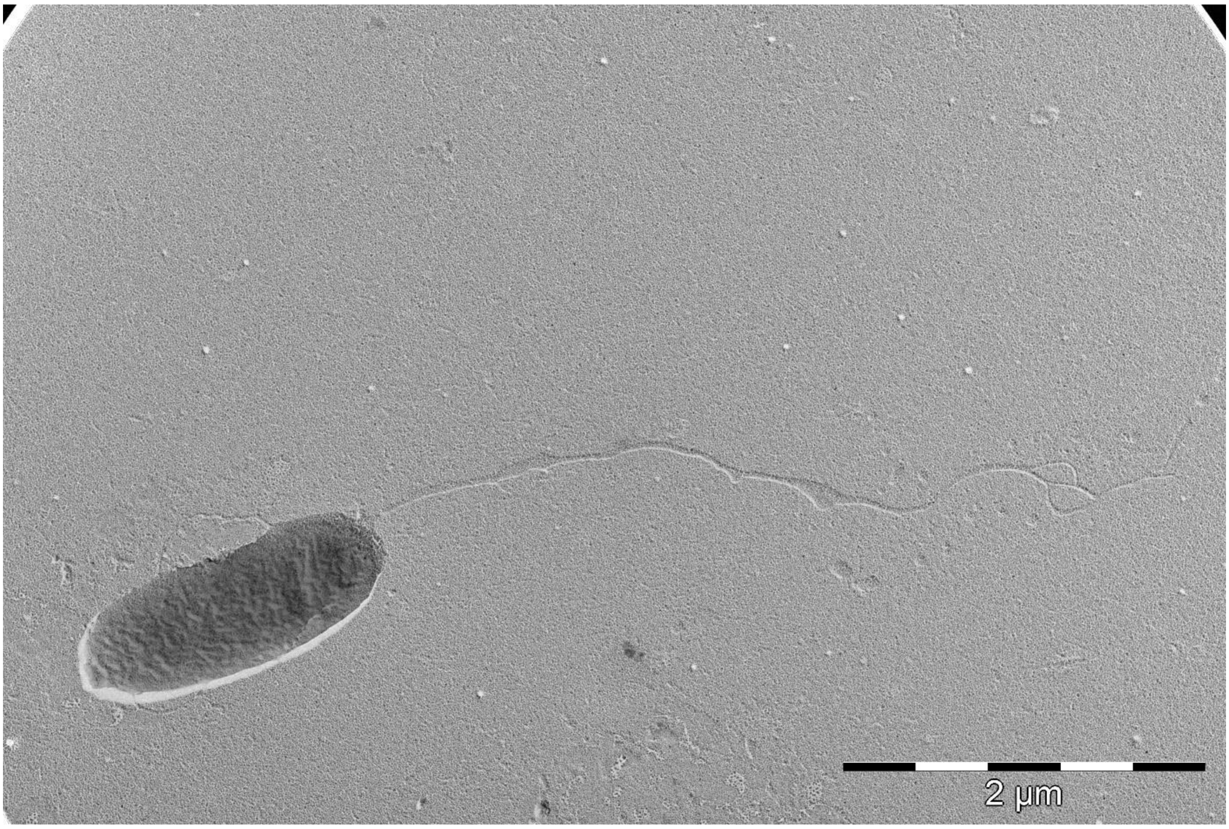

Figure S4. Transmission electron microscopy of *Ideonella benzenivorans* B7^T grown in R2A agar at 28ºC for 2-3 days. The bacterium is rod-shaped and motile with polar flagellum (using H-7100; Hitachi). Scale bar= 2 μm

DPG = Diphosphatidylglycerol

PE = Phosphatidylethanolamine

PG = Phosphatidylglycerol

APL = Aminophospholipid

AL = Aminolipid

L = Lipid

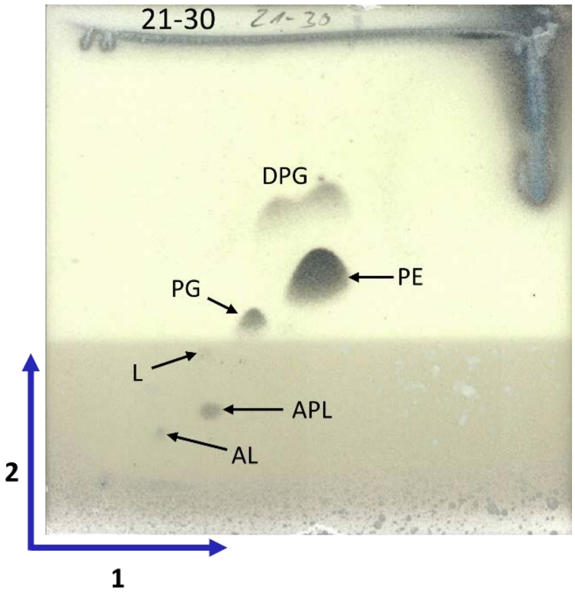

Figure S5. Polar lipid profile of strain B7^T
